# Gross cystic disease fluid protein 15 (GCDFP-15) expression in breast cancer subtypes

**DOI:** 10.1186/1471-2407-14-546

**Published:** 2014-07-28

**Authors:** Silvia Darb-Esfahani, Gunter von Minckwitz, Carsten Denkert, Beyhan Ataseven, Bernhard Högel, Keyur Mehta, Gabriele Kaltenecker, Thomas Rüdiger, Berit Pfitzner, Kornelia Kittel, Bettina Fiedler, Klaus Baumann, Roland Moll, Manfred Dietel, Holger Eidtmann, Christoph Thomssen, Sibylle Loibl

**Affiliations:** Institute of Pathology, Charité Universitätsmedizin Berlin, Charitéplatz 1, 10117 Berlin, Germany; German Breast Group (GBG Forschungs GmbH), Neu-Isenburg, Germany; University Women’s Hospital, Frankfurt am Main, Germany; Department of Gynecology and Obstetrics Rotkreuzklinikum München, Munich, Germany; Instititue of Pathology Rotkreuzklinikum München, Munich, Germany; Department of Gynecology and Obstetrics, Städtisches Klinikum Karlsruhe, Karlsruhe, Germany; Institute of Pathology, Städtisches Klinikum Karlsruhe, Karlsruhe, Germany; Praxisklinik Berlin, Berlin, Germany; Institute of Pathology, Sana Klinikum Lichtenberg, Berlin, Germany; Department of Gynecology and Obstetrics, University Hospital Giessen/Marburg, Marburg, Germany; Insitute of Pathology, University Hospital Giessen/Marburg, Marburg, Germany; Department of Gynecology and Obstetrics, Universitätsklinikum Schleswig-Holstein, Kiel, Germany; Department of Gynecology, Universitätsklinikum Halle (Saale), Halle (Saale), Germany

**Keywords:** GCDFP-15, Breast cancer, Neoadjuvant chemotherapy, Apocrine, CUP

## Abstract

**Background:**

Gross cystic disease fluid protein 15 (GCDFP-15), which is regulated by the androgen receptor (AR), is a diagnostic marker for mammary differentiation in histopathology. We determined the expression of GCDFP-15 in breast cancer subtypes, its potential prognostic and predictive value, as well as its relationship to AR expression.

**Methods:**

602 pre-therapeutic breast cancer core biopsies from the phase III randomized neoadjuvant GeparTrio trial (NCT00544765) were investigated for GCDFP-15 expression by immunohistochemistry. Expression data were correlated with disease-free (DFS) and overall survival (OS) time as well as pathological complete response (pCR) to neoadjuvant chemotherapy.

**Results:**

239 tumors (39.7%) were GCDFP-15 positive. GCDFP-15 expression was positively linked to hormone receptor (HR) and HER2 positive tumor type, while most triple negative carcinomas were negative (p < 0.0001). GCDFP-15 was also strongly correlated to AR expression (p 0.001), and to the so-called molecular apocrine subtype (HR-/AR+, p < 0.0001). Higher rates of GCDFP-15 positivity were seen in tumors of lower grade (<0.0001) and negative nodal status (p = 0.008). GCDFP-15 positive tumors tended to have a more favourable prognosis than GCDFP-15 negative tumors (DFS (p = 0.052) and OS (p = 0.044)), which was not independent from other factors in multivariate analysis. GCDFP-15 expression was not linked to pCR. Histological apocrine differentiation was frequent in molecular apocrine carcinomas (60.7%), and was associated with GCDFP-15 within this group (p = 0.039).

**Conclusions:**

GCDFP-15 expression is higher in tumors with favorable prognostic features. GCDFP-15 expression is further a frequent feature of AR positive tumors and the molecular apocrine subtype. It might have reduced sensitivity as a diagnostic marker for mammary differentiation in triple negative tumors as compared to HR or HER2 positive tumor types.

**Electronic supplementary material:**

The online version of this article (doi:10.1186/1471-2407-14-546) contains supplementary material, which is available to authorized users.

## Background

Gross cystic disease fluid protein 15 (GCDFP-15, syn. prolactin-inducible protein, PIP) is a 15 kDa protein that was originally detected in the cystic fluid from cystic mastopathy [1]. It is not expressed in normal ductal or lobular epithelium but in apocrine metaplasia of the breast [2]. Apart from breast cancer, only very few tumors, such as prostate cancer and carcinomas of the skin appendages express GCDFP-15 [3]. It is therefore highly specific for mammary differentiation in females, and is frequently used as an immunohistochemical marker for the evaluation of a potential mammary origin of metastatic carcinoma of unknown primary site. The expression of GCDFP-15 is regulated by the androgen receptor (AR) [4], however, little is known about its function. A recent study on gene expression profiles in androgen-stimulated, GDCFP-15 expressing versus GCDFP-15 non-expressing breast cancer cell lines, reported an up-regulation of pro-apoptotic and anti-proliferative genes along with GCDFP-15 [5]. In carcinomas of the breast, GCDFP-15 is also used as a marker for apocrine differentiation [2, 6–9]. Apocrine breast carcinoma is a rare subtype of invasive ductal carcinoma, which is primarily defined by morphological features such as abundant eosinophilic and granular cytoplasm, and shows frequent expression of the androgen receptor (AR) [10]. Some years ago a so-called molecular apocrine subset of breast carcinoma has been defined by gene expression analysis, and was characterized by active AR and weak or absent estrogen receptor (ER) signalling [11]. In this study, all tumors that were assigned to the molecular apocrine group had strong morphological features of apocrine differentiation. The existence of the molecular apocrine subtype has since then been reproduced [12, 13]. However, its clinical impact is conflictive to date.

We used a large and well-characterized cohort of breast cancer patients who underwent anthracycline/taxane-based neoadjuvant chemotherapy (NACT) in the phase III randomized GeparTrio trial (NCT00544765) to investigate the distribution of GCDFP-15 expression in biological subtypes of breast cancer, its potential prognostic and predictive value, as well as its relationship to AR expression. GCDFP-15 expression and biological tumor types were determined by immunohistochemistry in pre-therapeutic breast cancer core biopsies.

## Methods

### Study Population

Samples from the prospective neoadjuvant phase III GeparTrio study (NCT00544765) and the GeparTrio pilot study performed by the German Breast Group (GBG), Neu-Isenburg, Germany were used. Patients were treated with anthracycline/taxane-based NACT. The details of study setup and treatments have been published before [14–17]. HER2 positive patients had not received trastuzumab in GeparTrio as this was not the standard of care during the study period. Baseline clinico-pathological data as well as data on hormone receptor (HR) status were extracted from the study databases. Centrally evaluated data on HER2 expression (based on immunohistochemistry and silver-enhanced in situ hybridization according to ASCO/CAP guidelines [18] were used, as HER2 determination was not yet fully established in all pathologic laboratories at the time the study was conducted. Grading and histology were also centrally determined; local data on HR expression were used and substituted with central data if missing (central evaluation: Institute of Pathology, Charité Berlin). Consistent with the current practise when the trial was performed, HR positivity was defined as estrogen (ER) or progesterone receptor (PR) expression in more than 10% of tumor cells. We also exploratorily applied the cutoff of 1% currently recommended by ASCO/CAP [19]; use of this definition of HR positivity yielded quite similar results (see results section). Data on AR expression had been obtained from 545 cases in a previous study [20]. In brief, AR staining intensity as well as percentage of stained cells was multiplied to an immunoreactivity score (IRS), ranging from 0 (negative in all cells) to 12 (strongly expressed in more than 80% of cells). Cases with an IRS from 0–3 were scored as AR negative, opposed to cases with an IRS of 4–12 (=AR positive). Definition of pathological complete response (pCR) was complete absence of invasive tumour cells in the breast and lymph nodes as assessed at the time of surgery by the local pathologist (ypT0/Tis; ypN0). Data on disease-free survival (DSF) and overall survival (OS) were available for 570 patients, with mean DFS of 3.08 years and mean OS of 3.42 years. The baseline demographic and clinical characteristics of the patients with tissue available for this translational research project are shown in Table 1. The protocol was reviewed and approved by all responsible ethics committees (Additional file 1: Table S1). All patients provided written informed consent for anonymized subsequent translational research.

**Table 1:** **Association of GCDFP-15 expression with baseline clinico-pathological parameters**

	Total (100%)	GCDFP-15 negative	GCDFP-15 positive	p
Total	602	363 (60.3%)	239 (39.7%)	-
**HR** (n = 576)				0.034^a^
Negative	175	116 (66.3%)	59 (33.7%)	
Positive	401	228 (56.9%)	173 (43.1%)	
**HER2** (n = 576)				0.035^a^
Negative	478	299 (62.6%)	179 (37.4%)	
Positive	116	60 (51.7%)	56 (48.3%)	
**Biological tumor types** (n = 569)				0.001^b^
HR+/HER2-	328	189 (57.6%)	139 (42.4%)	
HR+/HER+	68	37 (54.4%)	31 (45.6%)	
HR-/HER2+	43	19 (44.2%)	24 (55.8%)	
HR-/HER2-	130	96 (73.8%)	34 (26.2%)	
**AR** (n = 545)				<0.0001^a^
Negative	238	180 (75.6%)	58 (24.4%)	
Positive	307	149 (48.5%)	158 (51.5%)	
**Age** (n = 602)				0.066^a^
< 50 years	273	176 (64.5%)	97 (35.5%)	
> = 50 years	329	187 (56.8%)	142 (43.2%)	
**Histological type** (n = 602)				0.090^b^
Ductal/others	554	340 (61.4%)	214 (38.6%)	
Lobular	48	23 (47.9%)	25 (52.1%)	
**Grading** (n = 601)				<0.0001^a^
G1-2	463	261 (56.4%)	202 (43.6%)	
G3	138	102 (73.9%)	36 (26.1%)	
**cT** (n = 591)				0.181^a^
cT1-2	395	230 (58.2%)	165 (41.8%)	
cT3-4	196	126 (64.3%)	70 (35.7%)	
**cN** (n = 583)				0.008^a^
cN0	261	141 (54.0%)	120 (46.0%)	
cN+	322	209 (64.9%)	113 (35.1%)	

^a^Fisher’s exact test.

^b^Pearson’s chi square test.

### Immunohistochemistry

Construction of a tissue micro array (TMA) of pre-therapeutic core biopsies has been explained previously [20]. Immunohistochemistry was performed on a Ventana BenchMark XT instrument (Ventana Medical Systems Inc., Tucson, AZ, USA) after pre-treatment with protease using a mouse monoclonal antibody directed against human GCDFP-15 (clone D6, dilution 1:400, Covance, Princeton, NJ, USA). For visualization, the iView DAB detection kit (Ventana Medical Systems Inc.) was used. Stained slides were digitized and evaluated on the computer monitor with support of the TMA Evaluator software (VMScope GmbH, Berlin, Germany) by a board certified pathologist (S. D.-E.). Both staining intensity and the percentage of stained tumor cells were evaluated and combined to an IRS (see previous chapter).

### Statistical evaluation

Statistical analysis was performed using SPSS Statistics 19 (IBM Corporation, Somers, NY, USA). In logistic regression analyses, significance of the correlation with pCR was assessed by the Wald test. Survival analyses were performed with the Kaplan-Meier method and univariate log-rank test and with Cox regression analysis for multivariate tests. The association between GCDFP-15 expression and clinico-pathological factors, biological tumor types, and AR expression was analysed by Fisher’s exact test or Pearson’s chi square test, as indicated. All tests were two-sided, and p-values <0.05 were considered as significant.

## Results

### GCDFP-15 expression pattern in human breast carcinomas

**Figure 1:**
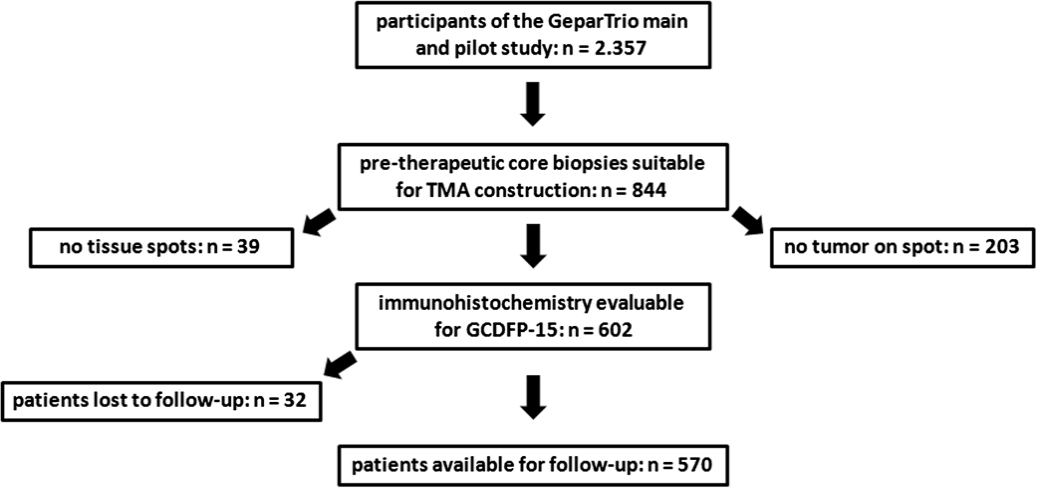
**Consort diagram.**

**Figure 2:**
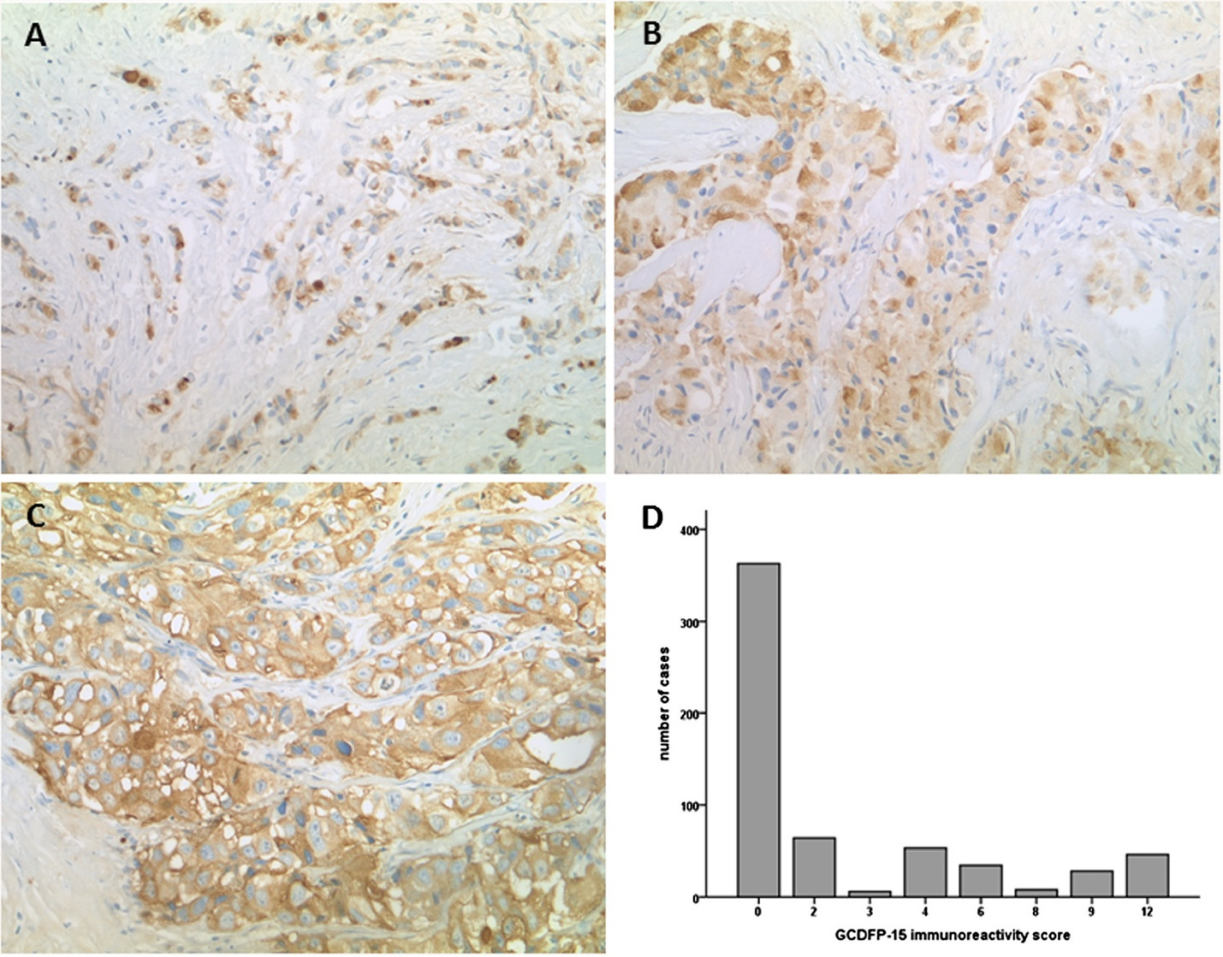
**Immunohistochemical expression pattern of GCDFP-15 in breast cancer. A** Tumor cells of an invasive lobular carcinoma, arranged in indian file pattern and exhibiting moderate cytoplasmic staining for GDCFP-15 **B** Solid carcinoma nests with patchy, mosaic-like pattern of GCDFP-15 expression **C** Diffuse GCDFP-15 expression in a poorly differentiated ductal carcinoma **D** Distribution of GCDFP-15 immunoreactivity scores (IRS) in the study group. The majority of cases did not display any staining (IRS = 0); in the remaining carcinomas, IRS values were equally distributed; the cut-off was set between IRS = 0 and IRS = 2.

844 TMA spots were evaluated (one for each individual tumor), whereas 203 spots contained no tumor cells (24.1%), and 39 spots contained no tissue (4.6%), resulting in 602 informative cases (Figure 1). Consistent with previous reports [21, 22], we found GCDPF-15 restricted to the cytoplasm of tumor cells. Positive tumors mostly displayed a weak to moderate stain in variable fractions of cells, and a patchy or mosaic-like pattern could be frequently found (Figure 2A, B). Sparse tumors showed diffuse staining (Figure 2C). In the majority of samples however, GCDFP-15 expression was totally absent (n = 363 (60.3%, Figure 2D)). We therefore decided to score each apparent staining as positive and opposed it to totally lacking staining. No GCDFP-15 staining was seen in non-epithelial cells such as stromal or inflammatory cells.

### Association with biological tumor types and clinico-pathological factors

GCDFP-15 expression was significantly enriched in tumors with certain biological characteristics. There was a modest increase of GDCFP-15 expression in HR and in HER2 positive tumors (Table 1). GCDFP-15 positivity rate was 43.1% in HR positive tumors and 33.7% in HR negative tumors (p = 0.034), and 48.3% HER2 positive carcinomas expressed GCDFP-15 as opposed to 37.4% HER2 negative tumors (p = 0.035). Consequently, GCDFP-15 was also differentially distributed among biological tumor types, as defined by HR and HER2 status: frequency of positive tumors was significantly higher in luminal subtypes (HR+/HER2-: 42.4%, HR+/HER2: 45.6%) as well in HER2 positive tumors (HR-/HER2+: 55.8%) as opposed to in triple negative breast carcinomas (TNBC, HR-/HER2-: 26.2%, p = 0.001, Table 1). Consistent with GCDFP-15 being a downstream target gene of AR [4], there was a strong association between GCDFP-15 and AR expression. 51.5% of AR positive tumors were also positive for GCDFP-15, whereas only 24% of AR negative carcinomas showed GCDFP-15 staining (p < 0.0001). As AR is a frequent feature of apocrine tumor differentiation and GCDFP-15 has also been proposed as a marker for apocrine differentiation, we tested GCDFP-15 expression for an association with the so-called molecular apocrine subtype (HR-/AR+), as described by Farmer et al. [11]. Tumor types according to Farmer were grouped as follows: HR + (AR+/-), n = 365), HR-/AR + (n = 56), and HR-/AR- (n = 101). HER2 positivity was more frequent in molecular apocrine carcinomas (42.9% as opposed to 17.1% in HR + (AR+/-) and 15.8% in HR-/AR-, p < 0.0001). There was a significant association between GCDFP-15 and molecular apocrine subtype, with 67.9% GCDFP-15 positive cases in this group (p < 0.0001). 41.6% of HR + (AR+/-) tumors were also positive for GCDFP-15, while the rate of GCDFP-15 positive cases in the subgroup that was completely negative for steroid hormone receptors (HR-/AR-) with 18.8% was lower than the one in triple negative tumors (HR-/HER2-, 26.2%, Table 1). GCDFP-15 was further associated with certain favorable tumor features, such as lower tumor grade (p < 0.0001), and negative nodal status (p = 0.008, Table 1). Using the currently by ASCO/CAP guidelines [19] recommended cutoff for ER/PR evaluation (<1% stained tumor cells = negative, > = 1% stained tumor cells = positive) we obtained similar results: HR positivity rate in the total study group was 79.5%, GCDFP-15 expression still was associated with HR positivity (p = 0.046) and with molecular apocrine tumor type (p < 0.0001), although the rate of molecular apocrine tumors was now decreased to 6.5%.

### Morphological features of molecular apocrine carcinomas

**Figure 3:**
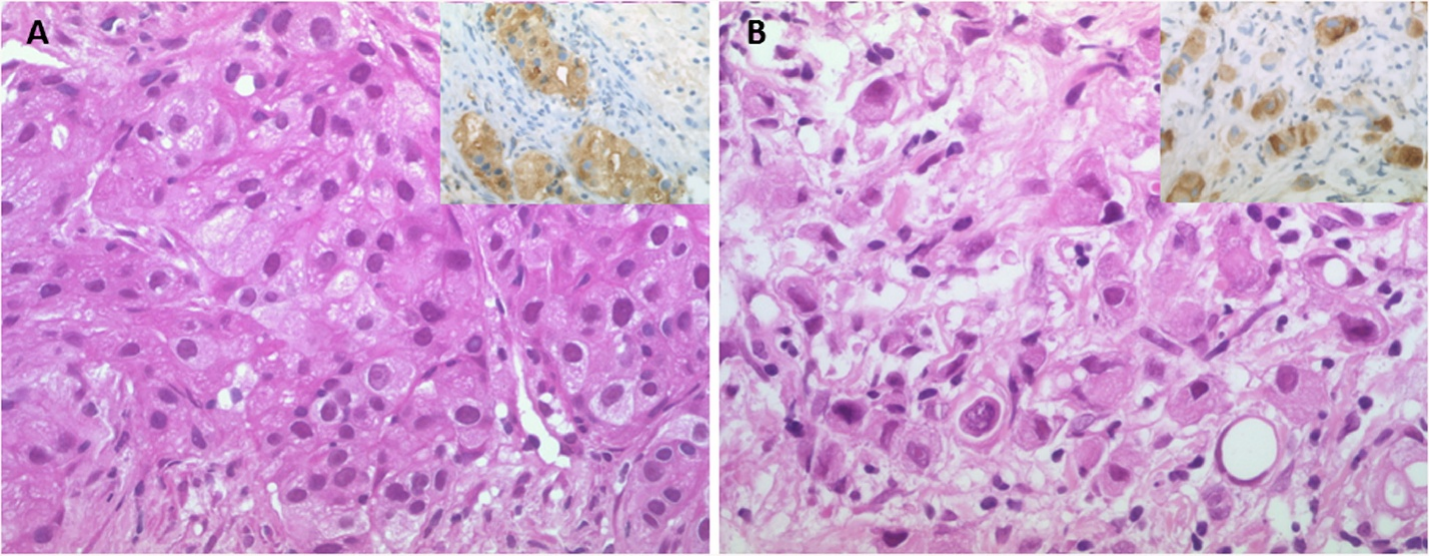
**Morphology of molecular apocrine carcinomas. A** Apocrine carcinoma with abundant eosinophilic granular cytoplasm exhibiting diffuse GCDFP-15 expression (insert) **B** Pleomorphic lobular carcinoma with dyscohesive growth of large cells with highly atypical nuclei and eosinophilic granular cytoplasm, strong diffuse GCDFP-15 expression is seen by immunohistochemistry (insert).

We further wondered whether the molecular apocrine subtype was showed a distinct morphology and re-evaluated hematoxylin/eosin-stained large sections of pre-therapeutic core biopsies according to apocrine differentiation. Criteria were based on Vranic et al. (2013) [6] and included tumors with large nuclei and characteristic abundant eosinophilic granular or foamy cytoplasm (type A, type B cells). Indeed, molecular apocrine carcinomas were quite frequently of apocrine phenotype (34/56, 60.7%, Figure 3A). Two carcinomas of pleomorphic lobular subtype, a poorly differentiated subgroup of lobular-invasive carcinoma reported to cluster with molecular apocrine tumors by gene expression analysis [23], were among them (Figure 3B). Histologically apocrine carcinomas with HR-/AR + profile were GCDFP-15 positive in most cases (27/34, 71.1%, p = 0.039; Figure 3A, B).

### Prognostic impact of GCDFP-15 expression

**Figure 4:**
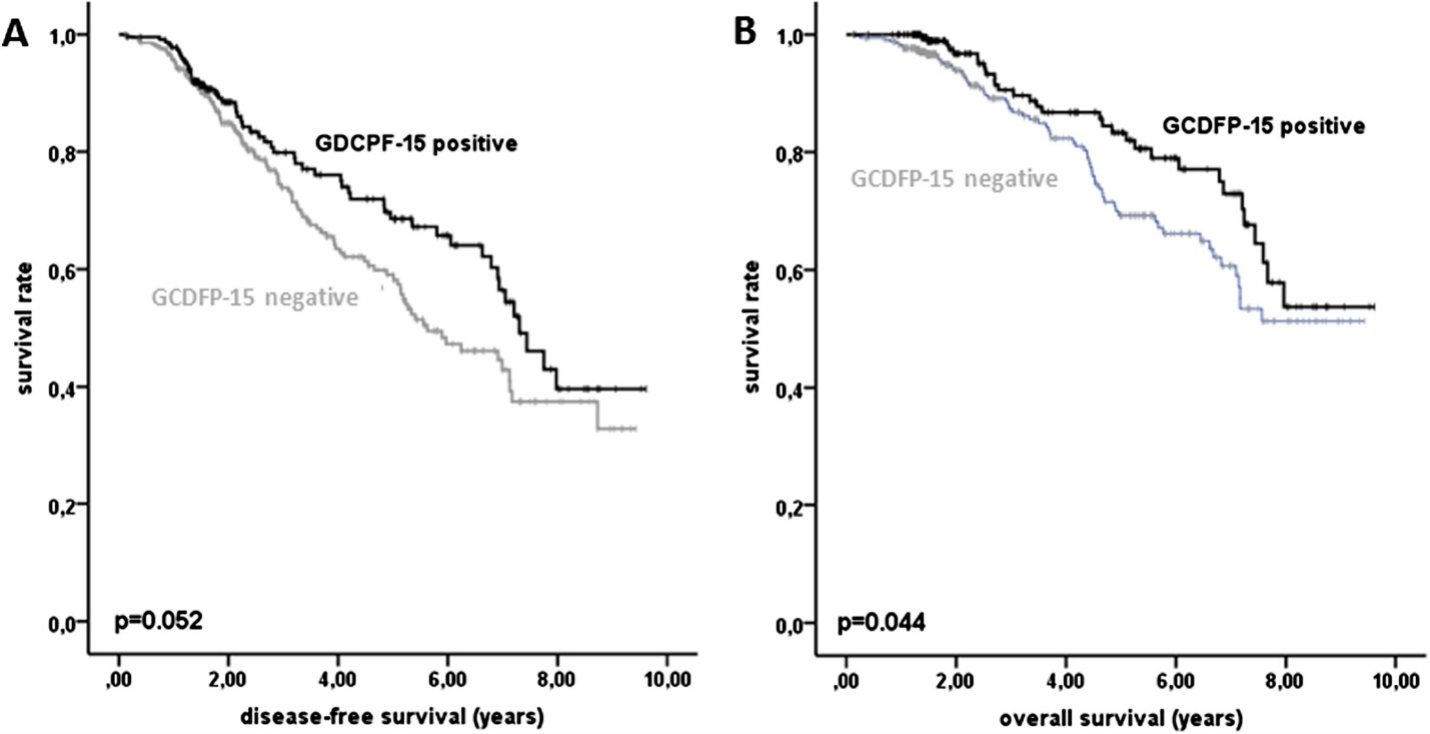
**Survival analysis A, B DFS and OS in dependence of GCDFP-15 expression in the study group.**

GCDFP-15 expression was also studied for a potential prognostic impact. GCDFP-15 positive tumors tended to have a more favourable prognosis than GCDFP-15 negative tumors (DFS (p = 0.052) and OS (p = 0.044)) in the study group (Figure 4A, B, Table 2). Explorative multivariate Cox regression analysis including HR and HER2 expression, age, nodal stage, and grading showed that GCDFP-15 expression was not an independent prognostic factor for OS (HR = 0.67, 95% CI = 0.37-1.20, p = 0.179, not shown). Similarly, GCDFP-15 was not a significant prognostic marker for OS or DFS within the biological tumor types (HR+/HER-, HR+/HER2+, HR-/HER2+, HR-/HER2-) or in Farmer tumor types (HR + (AR+/-), HR-/AR+, HR-/AR-; p < 0.05 for each test, not shown). Farmer tumor types by themselves were also prognostic for DSF and OS (log rank p = 0.024 for each), however a survival difference was seen only between HR- and HR + tumors, and was irrespective of an additional AR expression (data not shown). The following established prognostic makers for DFS and/or OS were significant in univariate analysis in the GeparTrio cohort as well: HR expression, biological tumor types, age, tumor grade, cT, and cN (Table 2).

**Table 2:** **Univariate survival analysis**

	DFS			OS		
	% events	Mean survival, years (SE)	p	% events	Mean survival, years (SE)	p
**GCDFP-15**						0.044
Negative	30.3	5.85 (0.24)		19.3	7.07 (0.23)	
Positive	25.1	6.67 (0.30)	0.052	13.9	7.75 (0.27)	
**HR**						0.013
Negative	35.6	5.53 (0.36)		23.3	6.75 (0.34)	
Positive	25.0	6.48 (0.23)	0.004	19.3	7.67 (0.34)	
**HER2**						0.686
Negative	26.3	6.31 (0.22)		16.0	7.46 (0.21)	
Positive	37.0	5.64 (0.40)	0.114	22.3	7.06 (0.24)	
**Biological tumor types**						0.084
HR+/HER2-	23.2	6.61 (0.25)		13.7	7.71 (0.24)	
HR+/HER2+	33.8	5.86 (0.52)		16.9	7.45 (0.43)	
HR-/HER2+	43.6	4.98 (0.60)		30.8	6.23 (0.53)	
HR-/HER2-	33.3	5.59 (0.42)	0.011	21.1	6.86 (0.41)	
**Age**						0.046
< 50 years	24.9	6.48 (0.28)		12.8	7.79 (0.25)	
> = 50 years	31.0	5.93 (0.26)	0.120	20.8	7.06 (0.24)	
**Histological type**						0.915
Ductal/others	28.8	6.15 (0.20)		17.2	7.40 (0.19)	
Lobular	10.0	6.42 (0.55)	0.309	17.4	6.82 (0.55)	
**grading**						0.043
G1-2	26.4	6.42 (0.22)		16.1	7.54 (0.20)	
G3	34.6	5.35 (0.41)	0.005	20.8	6.70 (0.40)	
**cT**						0.001
cT1-2	20.8	6.90 (0.24)		12.4	7.88 (0.22)	
cT3-4	43.9	4.93 (0.31)	<0.0001	26.7	6.65 (0.31)	
**cN**						<0.0001
cN0	22.4	6.91 (0.28)		11.4	8.12 (0.24)	
cN+	33.9	5.56 (0.26)	<0.0001	22.5	6.75 (0.25)	

DFS: disease-free survival.

OS: overall survival.

SE: standard error.

### Predictive value of GCDFP-15 expression

We further evaluated if GCDFP-15 expression might have predictive value for response to NACT and performed logistic regression analysis. In the total study group, there was a non-significant trend towards a reduced probability of pCR in GCDFP-15 positive tumors (pCR rate 21.2% vs 15.9%, OR = 0.70, 95% CI = 0.46-1.08, p = 0.106, Table 3). GCDFP15 expression was not indicative of response to NACT within biological tumor types or Farmer tumor types (p < 0.05 for each test, not shown). Farmer tumor types were significantly predictive for pCR, however similarly to the survival analysis, only the HR status was relevant for the predictive effect, and there was no difference between molecular apocrine (HR-/AR+) and HR-/AR- tumors: odds ratio (OR) of HR-/AR + 4.1 (95% CI 2.1-7.7), pCR rate 33.9%; OR of HR-/AR- 4.2 (95% CI 2.5-7.1), pCR rate 34.7%, as compared to HR+, respectively (p < 0.0001, pCR rate 11.2%). Already known predictive factors were also significant in our cohort: age, histological type, grade, HR expression, HER2 expression, and biological tumor types (Table 3).

**Table 3:** **Univariate logistic regression: association with pCR**

	n	Events	% pCR	OR	95% CI	p
**GCDFP-15**						0.106
Negative	363	77	21.2	1	-	
Positive	239	38	15.9	0.70	0.46-1.08	
**HR**						<0.0001
Negative	175	61	34.9	1	-	
Positive	401	45	11.2	0.24	0.15-0.37	
**HER2**						<0.0001
Negative	478	77	16.1	1	-	
Positive	116	36	31.0	2.34	1.48-3.72	
**Biological tumor types**						<0.0001
HR+/HER2-	328	28	8.5	1	-	
HER2+ (HR+/-)	111	34	30.6	4.73	2.70-8.28	
HR-/HER2-	130	43	33.1	5.30	3.11-0.02	
**Age**						0.001
< 50 years	273	68	24.9	1	-	
> = 50 years	329	47	14.3	0.50	0.33-0.76	
**Histological type**						
Ductal/others	554	113	20.4	1	-	
Lobular	48	2	4.2	0.17	0.04-0.71	
**Grading**						<0.0001
G1-2	463	74	16.0	1	-	
G3	138	41	29.7	2.22	1.43-3.46	
**cT**						0.098
cT1-2	395	83	21.0	1	-	
cT3-4	196	30	15.3	0.68	0.43-1.07	
**cN**						0.809
cN0	261	49	18.8	1	-	
cN+	322	63	19.6	1.05	0.70-1.59	

OR: Odd’s ratio.

CI: confidence interval.

## Discussion

We investigated the expression of GCDFP-15 in a large and well-characterized clinical trial cohort of breast carcinomas treated with NACT with a special emphasis on its distribution in breast cancer subtypes and its prognostic impact. We found that GCDFP-15 was increased in HR positive as well as in HER2 positive subtypes as compared to TNBC (HR-/HER2-). GCDFP-15 expression was not predictive for response to NACT. Although GCDFP-15 was a favorable prognostic factor for DFS and OS in univariate analysis, this impact was not independent from other factors and not evident within breast cancers subtypes. GCDFP-15 was furthermore strongly associated with AR and therefore enriched in the so-called molecular apocrine breast cancer subtype.

Although widely used as a diagnostic marker for breast carcinoma in pathology, the prognostic value of GCDFP-15 has not been systematically evaluated to date. Pagani (1994), in a small case series of 33 breast cancers found evidence of a longer relapse-free survival in patients with tumors positive for GCDFP15 gene expression [24]. Fritzsche et al. (2007) reported GCDFP-15 as a positive prognostic factor in a cohort of 165 carcinomas [25]. However, in this study the prognostic impact of GCDFP-15 expression was not investigated for each biological tumor type separately. Due to the very different molecular biology of those breast cancer subtypes, biomarkers may have quite varying prognostic implication within the subtypes. We show here that the prognostic impact of GCDFP-15 is most likely a bystander effect of its association with other factors, such as HR expression, nodal stage, and tumor grade. Our study thereby confirms previous findings of an association between GCDFP-15 expression and features of good-prognosis tumors [7, 22, 26], and it might be speculated that GCDFP-15 parallels the expression of its regulatory factor AR, which is also linked to favorable prognostic clinico-pathological features, as we showed previously [20]. We further found that GCDFP-15 is differentially expressed in breast cancer subtypes and is enriched in luminal and HER2 positive carcinomas, while being relatively sparse in TNBC. Similarly, Huo et al. (2013) reported a rather low percentage of GCDFP-15 positives in primary (14%) and metastatic TNBC (21%) [19]. Lewis et al. (2011) found even higher rates of GCDFP-15 expression than us in luminal (65-71%) and in HER2 positive carcinomas (64%), and found only one out 33 TNBC (basal-like and unclassified triple negative tumors) to be positive for GCDFP-15, however, their cohort being relatively small, might have underestimated the frequency of GCDFP-15 positivity in TNBC [22]. Taken together, these data warrant care if GCDFP-15 is used as a diagnostic marker for mammary differentiation of metastases of a cancer of unknown primary (CUP) because a significant proportion of breast cancers, particularly TNBC might be negative. An extended panel of immunohistochemical markers for mammary differentiation should be used to increase sensitivity. We show an enrichment of GCDFP15 expression in HER2 positive tumors and a strong association with AR expression, and are therefore in line with previous reports [7, 22, 27]. Not surprisingly, we further found GCDFP-15 to be elevated in the so-called molecular apocrine carcinomas that are defined by AR expression in the absence of HR expression [11]. In our study histological apocrine differentiation was found in 60.7% of molecular apocrine carcinomas; additionally, GCDFP-15 within the molecular apocrine subgroup was associated with histological signs of apocrine differentiation, which suggests that ER/PR, AR, and GCDFP-15 expression are helpful markers to confirm apocrine differentiation in morphologically conspicuous cases. On the other hand, 39.3% of HR-/AR + carcinomas did not show apocrine morphology in our cohort, which indicates that molecularly and morphologically defined apocrine groups overlap only partly. The clinical significance of the molecular apocrine subtype is not clear to date and remains to be determined as proposed by the current WHO Classification of Tumors of the Breast [28] (2012), similarly conflictive data exist regarding the prognostic impact of histologically defined apocrine subtype (reviewed by Vranic et al. [6]). Our study does not point to a particular prognosis or therapy response of HR-/AR + carcinomas, as pCR rate and survival times were quite similar to those in HR- tumors without AR expression. Interestingly, HER2 expression seems to interact with AR in HR negative tumors in prognostic terms, as in our previous study in the same cohort, AR positivity was a positive prognostic factor only in the molecular subgroup of triple negative breast cancer (as defined by ER/PR/HER2 negativity).

A limitation of our study is the reduced sample size in the HER2 positive tumor types (HR+/HER2+: n = 68, HR-/HER2+: n = 43), which might hamper the detection of a differential expression of GCDFP-15 or a prognostic impact of GCDFP-15 expression within those tumor types. Furthermore, relatively short follow-up times indicate that survival analysis should be interpreted cautiously (the GeparTrio study not being powered for survival as a primary end point). An additional limitation might be that we used a TMA constructed out of core biopsies, which in some cases contained only few tumor cells and which together with the focal expression pattern of GCDFP-15 might result in a reduced sensitivity for detection of GCDFP-15 expression in some tumors. However, the rate of GCDFP-15 expression in our study group (39.7%) was in the range reported in the literature [21, 26]. Only 602 out of 2.357 patients in the original GeparTrio studies could be included for this project; however this is still the largest study on GCDFP-15 expression to date.

## Conclusion

GCDFP-15 is expressed in all major biological breast cancer subtypes, and may be particularly useful as a diagnostic marker for mammary differentiation in HR and HER2 positive tumors, while there is reduced sensitivity in the triple negative subset. Due to its strong link to AR expression it may also be a marker for the so-called molecular apocrine subtype. GCDFP-15 is linked to clinico-pathological factors that indicate a better patient outcome, but is by itself no independent prognostic factor and is not predictive of response to anthracycline/taxane-based NACT.

## Electronic supplementary material

Additional file 1: **Ethics committees that approved the GeparTrio study.** (DOC 46 KB)
