# Supplementary material for: Gross cystic disease fluid protein 15 (GCDFP-15) expression in breast cancer subtypes
Source: BMC Cancer. 2014 Jul 28;14:546. doi: 10.1186/1471-2407-14-546 (PMC4122770; doi:10.1186/1471-2407-14-546)
Supplement: Supplementary file 1 — Additional file 1: Ethics committees that approved the GeparTrio study. (DOC 46 KB) [file 12885_2013_4721_MOESM1_ESM.doc]

**Supplemental table S1** Ethics committees that approved the GeparTrio study

| Ethics committee of the medical association of Berlin |
| --- |
| Ethics committee of the medical association of Hamburg |
| Ethics committee of the Medical Faculty of the University of Greifswald |
| Ethics committee of the medical association of Lower Saxony |
| Ethics committee of the medical association of [North](https://dict.leo.org/#/search=North&searchLoc=0&resultOrder=basic&multiwordShowSingle=on) [Rhine-Westphalia](https://dict.leo.org/#/search=Rhine-Westphalia&searchLoc=0&resultOrder=basic&multiwordShowSingle=on) |
| Ethics committee of the medical association of Saarland |
| | Ethics committee of the medical association of | [Saxony-Anhalt](https://dict.leo.org/#/search=Saxony-Anhalt&searchLoc=0&resultOrder=basic&multiwordShowSingle=on) | | --- | --- | |
| |  | Ethics committee of the medical association of Westphalia-Lippe and the Medical Faculty of the University of Münster | | --- | --- | |
| Ethics committee of the Bavarian medical association |
| Ethics committee of the medical association of the Medical Faculty of the Charité Berlin |
| Ethics committee II of the University of Heidelberg and the University Hospital Mannheim |
| Ethics committee of the medical association of Baden-Württemberg |
| Ethics committee of the medical association of Hessen |
| |  | Ethics committee of the medical association of [Rhineland-Palatinate](https://dict.leo.org/#/search=Rhineland-Palatinate&searchLoc=0&resultOrder=basic&multiwordShowSingle=on) | | --- | --- | |
| | Ethics committee of the medical association of | [Thuringia](https://dict.leo.org/#/search=Thuringia&searchLoc=0&resultOrder=basic&multiwordShowSingle=on) | | --- | --- | |
| Ethics committee of the medical association of Brandenburg |
| Ethics committee of the Medical Faculty of the Ludwig-Maximilians University Munich |
| Ethics committee of the medical association of Saxonia |
| Ethics committee of the Medical Faculty of the Johann-Wolfgang-Goethe University of Frankfurt am Main |
| Ethics committee of the Medical Faculty of the Georg-August University Göttingen |
| Ethics committee of the Medical Faculty of the Martin-Luther University Halle-Wittenberg |
| Ethics committee of the University of Ulm |
| Ethics committee of the Medical Faculty of the University of Lübeck |
| Ethics committee of the Medical Faculty of the Christian-Albrechts University of Kiel |
| Ethics committee of the Medical Faculty of the Otto-von-Guericke University of Magdeburg |
| Ethics committee of the Medical Faculty of the Eberhard-Karls University of Tübingen |
